# A Young Scientists’ Perspective on DBS: A Plea for an International DBS Organization

**DOI:** 10.1007/s12152-015-9231-x

**Published:** 2015-04-13

**Authors:** Rowan P. Sommers, Roy Dings, Koen I. Neijenhuijs, Hannah Andringa, Sebastian Arts, Daphne van de Bult, Laura Klockenbusch, Emiel Wanningen, Leon C. de Bruin, Pim F. G. Haselager

**Affiliations:** 1 Department of Philosophy, Radboud University Nijmegen, Erasmusplein 1, 6500 HD Nijmegen, The Netherlands; 2 Behavioural Science Institute, Radboud University Nijmegen, SP A.09.17, Spinozagebouw, Montessorilaan 3, P.O. Box 9104, 6525 HR Nijmegen, The Netherlands; 3 Radboud University Medical Centre, Radboud University Nijmegen, Geert Grooteplein-Zuid 10, 6525 GA Nijmegen, The Netherlands; 4 Political Science Department, Radboud University Nijmegen, Thomas van Aquinostraat 5, Postbus 9108, 6500 HK Nijmegen, The Netherlands; 5 Economics and Business Economics Department, Radboud University Nijmegen, Thomas van Aquinostraat 5, 6500 HK Nijmegen, The Netherlands; 6 Donders Institute for Brain, Cognition and Behaviour, Radboud University Nijmegen, B.01.13 Spinozagebouw, Montessorilaan 3, 6525 HR Nijmegen, The Netherlands

**Keywords:** Deep Brain Stimulation, Repository, Communication, Small-N studies

## Abstract

Our think tank tasked by the Dutch Health Council, consisting of Radboud University Nijmegen Honours Academy students with various backgrounds, investigated the implications of Deep Brain Stimulation (DBS) for psychiatric patients. During this investigation, a number of methodological, ethical and societal difficulties were identified. We consider these difficulties to be a reflection of a still fragmented field of research that can be overcome with improved organization and communication. To this effect, we suggest that it would be useful to found a centralized DBS organization. Such an organization makes it possible to 1) set up and maintain a repository, 2) facilitate DBS studies with a larger sample size, 3) improve communication amongst researchers, clinicians and ethical committees, and 4) improve communication between DBS experts and the public at large.

The Health Council of the Netherlands asked the think tank ‘Wider Implications of Cognitive Neuroscience’ of the Radboud University Nijmegen Honours Academy to investigate the implications of Deep Brain Stimulation (DBS) for psychiatric patients. The think tank consisted of a team of master students with backgrounds in various disciplines: philosophy, behavioral science, political science, medicine, and economics. In this brief communiqué, we present the outcomes of our investigation – not as categorical statements, but as the suggestions of a group of aspiring scientists that took a fresh look at this relatively new, important, and rapidly developing research area.^1^ The main conclusion of our report is that the establishment of a centralized organization of DBS experts is an important first step in addressing a number of methodological, ethical, and societal issues surrounding DBS. We will discuss four advantages of such an organization: a prospect of setting up and maintaining a repository, facilitating studies with a larger sample size, improving communication amongst researchers, clinicians and ethical committees, and improving communication between DBS experts and the public at large. We will end by arguing why existing organizations do not yet appear to be up to this task.

Investigations of DBS as a treatment for psychiatric disorders are still at an early stage. Currently, only treatment-refractory psychiatric patients are allowed DBS-treatment, which in turn results in small sample sizes [e.g., [1]]. Small sample sizes are customary during early phases of clinical trials [2]. For invasive treatments, these early phases often comprise samples of patients that are treatment-refractory. Because of the ethical concerns surrounding invasive techniques for non-neurodegenerative psychiatric illnesses,^2^ small sample trials may turn out to remain the status quo. Unfortunately, due to these small sample sizes, crucial control conditions are often missing. As the field may suffer from fragmentation due to many small studies, the creation of a repository has been proposed by numerous authors [3–6]. We were not able, however, to find any evidence for the existence of such a repository or its development. An important incentive for establishing a centralized organization is that setting up and monitoring a repository is no small feat and requires a coordination of efforts that existing (local) organizations may not be able to provide.

Additional benefits of a repository include transparency and the facilitation of communication between research groups. Transparency may be especially important due to possible conflicts of interests that may arise due to investment from a relatively small number of commercial companies in research [7]. Lastly, another benefit of a repository is being able to find (unpublished) null results; important in itself but even more crucial in the field of DBS where decisions need to be made about which patient groups are the most likely candidates to receive DBS.

Establishing and maintaining a repository is one way to mitigate the problem of small sample sizes. However, a centralized organization could mitigate the problem of small sample sizes in a second way. Instead of relying on multiple small studies that each employ slightly different methods, a number of (international) research groups may come together and set up larger multicenter studies of efficacy with a standardized research methodology. Setting up such large-scale studies not only requires plentiful resources but also a large and well-functioning infrastructure. A centralized organization could be able to provide this.

Aside from the methodological issues discussed above, the use of DBS faces a number of particularly unique ethical challenges. Some examples are personality or identity changes [8–13], informed consent [8, 14], and the use of DBS for enhancement [15]. Such ethical challenges require debate as well as expert guidance, both of which can be facilitated by establishing expert ethical committees (ECs) committed to including DBS experts. Regular ethical committees may not be sufficiently specialized in the aforementioned ethical challenges unique to DBS. An expert EC could advise university and institute ECs on the peculiarities and specificities of DBS research. A second role of an expert EC may be in advising hospitals and institutes regarding eligible patient groups. The proposed centralized organization could house such an expert EC and provide it with a communication infrastructure.

The general public is mostly informed of DBS through the media. The media, however, often portrays DBS too optimistically [16–18]. This bias may have both large- and small-scale implications. On the larger scale, the public may develop a distorted understanding of the technique, its purpose, its applications, and its effects. Such a distorted understanding may lead to a diminished public awareness of the technique, its uses, its risks, and its consequences. On the smaller scale, as patients often turn to the media to educate themselves about their condition and possible treatments [19, 20], they may already have a positively-biased opinion of the use of DBS before coming into contact with a clinician.

It is of great importance to establish a continuous dialogue between those working in the field of DBS and the general public. Such a dialogue may serve to inform the general public about the current state of the art in order to create realistic expectations regarding the efficacy of DBS. Additionally, it may facilitate better-informed ethical debate in the public sphere, for example, by organizing conferences for journalists, creating a website containing up-to-date information, and developing standardized communication procedures for researchers and research institutes for contact with the media. As this is a demanding task, a centralized organization could prove useful in realizing these kinds of implementations.

We mentioned above the benefit for communication by establishing a repository. It is worth noting that providing data and articles in a repository is a form of passive communication: people who are interested are able to look up relevant information. However, in addition to passive communication we argue for the facilitation of active communication, as it is important to keep relevant parties informed of new developments. Currently, this is partly achieved by DBS-specific and more general neurostimulation conferences. A centralized organization could facilitate the application of such active communication efforts by providing the necessary infrastructure.

As the topics we have discussed in this paper illustrate, many of the issues concerning DBS are the result of – in itself understandable – small-scale studies, leading to a fragmentation of the field and a lack of coherent communication between DBS experts and a wider audience. Due to the negative consequences of fragmentation, as discussed above, it is of importance that an organization encompasses all of the roles we have identified. These roles include maintaining a repository, housing an expert ethical committee, creating infrastructure for multicenter studies, facilitating communication among researchers and between researchers and the general public. While there are a number of international DBS organizations, these organizations are neither as specialized nor as all-inclusive as we would recommend. For example, the Dana Foundation’s mission states: “The Dana Foundation is committed to advancing brain research and to educating the public in a responsible manner about the potential of research” [21]. While this mission is close to our recommendations – concerning communication between professionals and the general public – this organization is not specialized to the extent of being able to, for example, provide an expert ethical committee or govern a repository. Another example would be the European Association of Neurosurgical Societies (EANS) [22]. While the EANS is specialized and invested in neurosurgery, neurotechnology is a specialization in its own right. Additionally, the EANS is less concerned with communication between experts and the general population than we see as necessary. A possible solution would be for one of the existing organizations to incorporate the responsibilities we identified but moves towards this are not yet being carried out. Another possibility is to form a completely new organization, preferably funded by international, governmental institutions like the European Union and supervised by a board of leading experts.

In conclusion, as young developing scientists new to the field, we present our observations as mere suggestions that, hopefully, are of interest to the experts. We neither have the experience nor the affiliations required to found the suggested organization, however to stimulate the debate we have identified a number of issues pertaining to the fragmentation of the field of DBS. We suggest that there is a need for a centralized organization as an important first step in reducing fragmentation in order to alleviate the methodological, ethical, and societal problems that DBS gives rise to.

## Footnotes

1 In this article we are only able to give a partial summary of the entire report. For a more thorough discussion, see our full report [23].

2 Generally, later stages could comprise larger groups of patients. For example, DBS has seen moderate success in the larger patient population with early indications of Parkinson’s Disease [24] and Alzheimer’s Disease [25]. However, it is debatable whether the use of DBS for neurodegenerative illnesses such as Parkinson’s is comparable to the use of DBS for non-neurodegenerative psychiatric illnesses. While Parkinson’s disease inevitably reaches the point of being untreatable, this is not the case for all psychiatric illnesses. As such, an ethical question that arises is whether an invasive technique such as DBS should be tested on psychiatric patients who have noninvasive treatment options left.
